# Patterns of care-seeking for postpartum symptoms in urban Karachi, Pakistan: implications for intervention design

**DOI:** 10.1186/s12978-025-01981-8

**Published:** 2025-04-16

**Authors:** Farzeen Hirani, Shabina Ariff, Apsara Ali Nathwani, Ghazal Peerwani, Anna Kalbarczyk, Shazia Sultana, Abdul Momin Kazi, Farheen Yousuf, Amnesty E. Lefevre, Shereen Bhutta, Peter J. Winch, Sajid Soofi, Zulfiqar A. Bhutta, Anita K. M. Zaidi, Fatima Mir, the ANISA-Postpartum Sepsis Study Group, Abdul Momin Kazi, Amnesty E. Lefevre, Sajid Soofi, Abdullah H. Baqui, Aneeta Hotwani, Anita K. Zaidi, Furqan Kabir, Iftekhar Rafiqullah, Megan E. Reller, Mohammad Shahidul Islam, Nicholas E. Connor, Sadia Shakoor, Samir K. Saha, Shahida M. Qureshi, Shams El Arifeen, Zulfiqar A. Bhutta Zaidi, Linda Bartlett

**Affiliations:** 1 Pediatrics and Child Health, The Aga Khan University, Karachi, Pakistan; 2 Department of International Health, Johns Hopkins Bloomberg School of Public Health, Baltimore, MD USA; 3 Obstetrics and Gynecology, The Aga Khan University, Karachi, Pakistan; 4 Obstetrics and Gynecology, Jinnah Postgraduate Medical Center, Karachi, Pakistan

**Keywords:** Postpartum sepsis, Recently delivered women, Care-seeking, Qualitative analysis, Pakistan

## Abstract

**Background:**

In Pakistan, the maternal mortality rate is 186/100,000 live births, with postpartum (PP) or maternal sepsis being the third leading cause of maternal deaths. Delays in early identification and timely management of PP sepsis are associated with mortality and severe maternal outcomes, including septicemia, neonatal deaths, infertility, etc. In this study, we aim to explore patterns of care-seeking of maternal health services by recently delivered women (RDW) in semi-urban Karachi, Pakistan. Insights of this study will help in identifying and addressing the barriers in care-seeking to minimize delay to improve clinical outcomes.

**Methods:**

We conducted 32 semi-structured qualitative interviews with RDW with PP sepsis, traditional birth attendants (TBAs), health care providers, and family relatives of RDW to characterize the patterns of care-seeking behaviors, sources of care, and treatment modalities. Community interviews were conducted in Bilal Colony, an urban squatter settlement, and facility interviews were conducted at a high-volume tertiary care facility in Karachi, Pakistan. All interviews were conducted face to face by trained data collectors which were then audio recorded. A codebook was developed manually by reviewing all transcripts and identifying emerging themes. Coded transcripts were entered into NVivo software to develop quotation summaries and models that identified subthemes.

**Results:**

This study utilized a 3-delay model to determine care utilization in RDW with PP sepsis. Phase 1 indicated limited awareness about PP symptoms, cultural norms, and lack of decision autonomy led to delayed care-seeking, as women depended on male or older female relatives for approval. Two of the most common symptoms of PP sepsis were high-grade fever and foul-smelling discharge, which were deemed as non-severe. Phase 2 findings implied that women initially sought care from TBAs, chemists, and faith healers, or self-medication and tertiary care was their last resort. Financial constraints were also determining care-seeking; Phase 3 indicated that women who sought care at the hospitals were in critical conditions due to prior unskilled care or traditional treatment choices.

**Conclusion:**

Increasing awareness of PP sepsis and its symptoms via educational programs is essential for not only women but also their family members who play roles in decision-making, Training community health workers and TBAs to recognize signs of PP sepsis and promptly refer women to appropriate facilities could also significantly reduce reliance on inappropriate care sources and ensure timely treatment.

**Supplementary Information:**

The online version contains supplementary material available at 10.1186/s12978-025-01981-8.

## Plain language summary

Postpartum (PP) sepsis is one of the serious complications after pregnancy resulting from infection of the genital tract occurring at labor or within 42 days of the PP period. Severe cases can lead to organ dysfunction and, ultimately, death. It can also be transferred to newborns, causing early-onset sepsis. PP sepsis is one of the leading causes of death in Pakistan in both hospitals and community settings. These deaths can be prevented by timely care-seeking and treatment. In this study, we explored care-seeking patterns in women who recently delivered in semi-urban Karachi, Pakistan. We conducted semi-structured interviews of recently delivered women (RDW) with PP sepsis, their families, traditional birth attendants, and physicians from the community and a tertiary care hospital. Only physicians were aware of PP sepsis. RDW, their families, and traditional birth attendants were aware of PP complications but couldn’t identify PP sepsis symptoms and were not aware of its severity. One of the major reasons highlighted for the delay in care seeking was that the women were not autonomous in their decisions to visit healthcare facilities and seek care. Husbands and relatives were responsible for decision-making, and their unavailability caused further delay. Trust in services due to previous experience, financial constraints, use of alternate medicine, limited reach, and lack of knowledge were other reasons for not seeking early care. If community healthcare workers are aptly trained to identify early signs of PP sepsis and for timely referral, it can aid in preventing further PP sepsis consequences.

**Supplementary Information:**

The online version contains supplementary material available at 10.1186/s12978-025-01981-8.

## Background

Postpartum (PP) or puerperal sepsis is defined as the infection of the genital tract occurring at labor or within 42 days of the PP period [1]. It is encompassed within the wider term maternal sepsis, which is the third leading cause of maternal mortality and is a life-threatening condition that arises as a response to infection, causing tissue damage and organ dysfunction during pregnancy, childbirth, post-abortion, and PP period [2]. As per the recent Global Burden of Disease in 2019, annually, there are 21 million incident cases of maternal sepsis and related maternal infections, culminating in 17,000 deaths worldwide [3]. Another study reported that approximately 11% of maternal deaths are attributed to PP sepsis [4]. The incidence of PP sepsis has shown vast variations in geographical regions around the world, with middle and lower-middle-income countries having the largest number of incident cases [5, 6]. Furthermore, it was also found that South Asia, along with a few countries of Africa, suffered an upward trend of maternal sepsis and related infections over the last few decades, which can be due to socioeconomic reasons: medical accessibility and other cultural constraints [5]. Moreover, case fatality rates were also highest in Southeast Asia (14.3%) and Africa (11.4%), which raises an alarm for immediate attention to prevent PP sepsis-related mortality and severe morbidity, specifically in these two regions [7].

PP sepsis has serious complications on not only maternal health but also neonatal wellbeing. Globally, one million neonatal deaths every year are attributed to maternal sepsis [5, 8]. Additionally, other maternal complications include septicemia, endotoxic shock, peritonitis, or abscess formation leading to surgery and death. Long-term consequences include chronic pelvic inflammatory disease, ectopic pregnancies, and infertility. It has been reported that 11/1000 women with PP sepsis or suspected infections suffer from severe maternal outcomes, averaging the case fatality rate to 6.8%, which increases in low-middle-income countries (LMICs) to 15%, with every 15/1000 women having severe maternal complications due to sepsis or death [4]. Pakistan is one such LMIC with resource-limited settings and health infrastructure not well equipped to cater to the high burden of obstetric complications. As per the Maternal Mortality Survey 2019, the maternal mortality rate in Pakistan is 186 deaths/100,000 live births, of which the majority (96%) were direct maternal deaths, implying that they were due to obstetric complications during pregnancy, labor, or within 42 days of delivery [9]. PP sepsis ranked the third major reason for maternal deaths, followed by obstetric hemorrhage and hypertensive disorders [10, 11]. However, these figures may underestimate the true impact, as a significant portion (28–42%) of births, especially in rural areas, occur at home with unskilled or traditional birth attendants, making data collection on maternal and neonatal mortality challenging [12].

There are National sepsis guidelines for Pakistan specifically designed and curated to aid in the early identification and management of sepsis in adults in local settings [13]. However, their implementation for the prevention and timely treatment of maternal sepsis in low-resource settings is still questionable. The high incidence of PP sepsis and mortality can be majorly due to delays in their detection and appropriate management. Identifying illness during the PP period is an important indicator of maternal disease burden, but the perception of and response to symptoms are equally important. The “Three Delays” model of maternal mortality by Thaddeus and Maine highlights the role of the health system and the community in maternal deaths. The three delays that result in maternal deaths are (1) delay in deciding to seek care, (2) delay in reaching the healthcare facility, and (3) delay in initiation of treatment after arrival [14]. Delay in seeking care during the PP period can hugely determine the entire course of identification and management of the sepsis. The most common reasons of delay in care-seeking for maternal health services include limited knowledge about maternal care, lack of education, perceived severity, lack of autonomy of women and male domination, religious and traditional belief systems, influence of spiritual healers, financial constraints, availability of transport, and self-medication [15–17].

Despite some advancements, maternal health knowledge and service utilization remain low in Pakistan, contributing to high maternal mortality and hindering the achievement of Sustainable Development Goal 3.1, which aims to reduce the maternal mortality ratio to below 70 per 100,000 live births [18]. Reducing maternal mortality from postpartum sepsis requires timely identification and treatment, which is only possible through early care-seeking by mothers. This formative study was undertaken as part of a larger study on post-partum sepsis in which we sought to develop and test a diagnostic algorithm for community health workers (CHWs) to identify PP women with symptoms of PP sepsis [19]. In this component of the study, we aimed to understand the patterns of care-seeking behaviors to improve timely and appropriate referrals for recently delivered women (RDW) with suspected infection in semi-urban Karachi, Pakistan.

## Methods

We conducted 32 in-depth interviews (IDIs) at a medical facility and a community site in Karachi, the largest city in Pakistan, from 6 August to 15 September 2012. Community interviews were conducted in Bilal Colony, an urban squatter settlement located in the Karachi industrial area where the leather industry is a dominant business. The majority of the population lives in small houses with 1 or a maximum of two bedrooms. A very high percentage of low or lower-middle-income families with limited formal education reside in this area. Cultural norms dictating heavy reliance on faith healers and traditional birth attendants/ health workers are common here. This study site will offer insight into the early stages of care-seeking by women in such small peri-urban settlements with limited healthcare access. Facility interviews were conducted at the Jinnah Postgraduate Medical Centre (JPMC) (Supplementary Fig. 1). This tertiary care facility is a high-volume referral center for all obstetric and gynecological emergencies and inpatient services for both the city of Karachi and most of the Sindh Province. Along with being high referral, this facility provides comprehensive care to patients across all socioeconomic backgrounds by offering affordable treatment and management options. Specializing in high-risk and complex cases, such as postpartum (PP) sepsis, the healthcare providers here are well-equipped with the expertise needed to handle such critical conditions. Participants included from the facility, included 6 healthcare workers, 4 RDWs, and 4 mother-in-law/ female relatives of RDW. A total of 14 participants were enrolled from the facility. In community, 6 TBA’s, 6 RDW and 6 mother-in-law/female relatives were enrolled making a total of 18 participants. A total of 10 RDWs, 10 mothers-in-law, 6 trained healthcare providers, and 6 TBAs were a part of this study.

Facility-based interviews were conducted with healthcare providers who had experience in diagnosing PP sepsis and providing maternal PP care. RDW were admitted to the facility and not incapacitated by any other illness so they could provide informed consent. Trational Birth Attendants (TBAs) were visited every week in the Bilal colony to identify possible PP sepsis cases. Family members were present in the household during the woman’s illness and participated in the care-seeking decision. Senior family members selected from community sites were mothers or mothers-in-law who had been in frequent contact with a woman during the PP period. TBAs and Community Health Workers (CHWs) had provided care for a woman with PP sepsis at least once. Supplementary Table 1 presents the demographic data for participants at both sites.

All interviews were conducted face-to-face (in-person) by trained data collectors in Urdu, the national language of Pakistan. Interview sessions were accompanied by a recorder who took notes and audio recorded the session. Recordings were transcribed in both Urdu and English. Interviews were conducted in separate rooms to ensure privacy and prevent male participation and external input. Each interview lasted from 35 to 60 min. RDW and their family members were asked to discuss where they sought care and the reasoning behind their choices. Our definition of care was not restricted to services provided by TBAs or physicians. Rather, we included self-medication (e.g., antibiotics, pain medications, solutions from unlicensed providers), faith healing, and any other sources identified by participants. Interview tools for TBAs and physicians included questions about perceived causes of delays in care and possible solutions (Supplementary file).

**Fig. 1:**
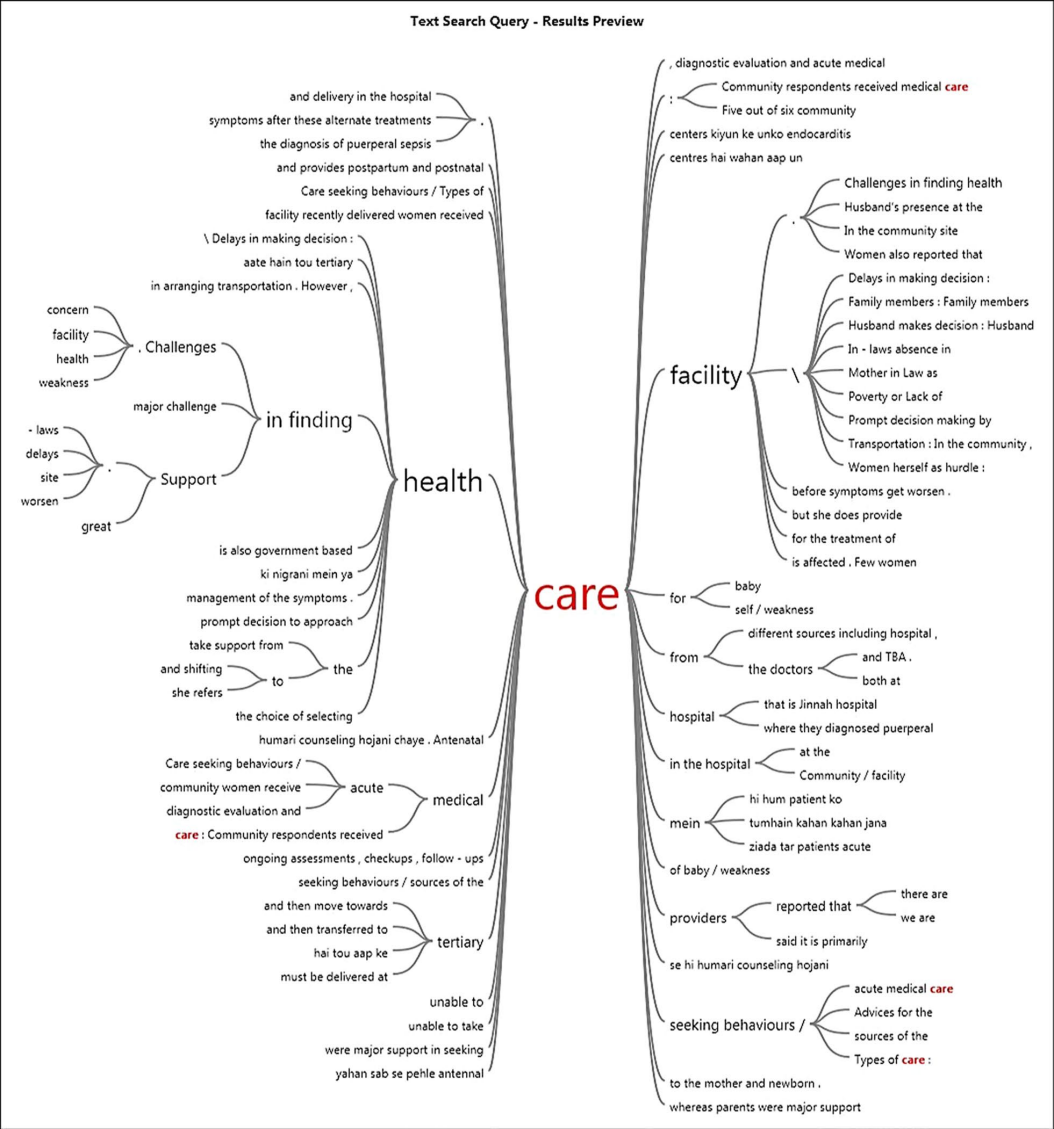
NVivo model of care and its subthemes

A codebook was developed manually by reviewing all transcripts and identifying emerging themes. Transcripts were entered into NVivo software, and the transcripts were then coded into themes. A query was generated to develop quotation summaries that identified subthemes. We used the visualization feature of NVivo to construct a model that depicts themes and subthemes. Figure 1 illustrates an example of the model developed for the theme “care”.

## Results

Using the 3-Delays Model, we built a framework to demonstrate the factors that affect service utilization and their relationship to the three phases of delay.

### Phase I: decision to seek care

#### Lack of knowledge of PP sepsis

Of all study participants, only physicians were aware of the term PP sepsis in part because no literal translation for the illness exists in Urdu, the national language of Pakistan. RDW and their female relatives were able to identify common postpartum illness symptoms but were not very aware of their significance. Furthermore, they could not differentiate symptoms of sepsis from other non-infectious complications of the PP period.

TBAs were much more aware of non-infectious post-partum complications, such as PP hemorrhage and retained placenta, and knew that these conditions required immediate referral.

#### Lack of attention to symptoms

High-grade fever and foul-smelling discharge, two common symptoms of PP sepsis, were locally managed and generally not taken as warning signs of a serious infection. Neither the family nor TBAs take these symptoms seriously enough to seek further care or referral.

#### The decision makers

In many South Asian countries, women are not the primary decision-makers regarding their health. They often need to acquire permission from senior female relatives and their husbands. All of our RDW participants reported that they were not autonomous in their decision to seek care. For two of the four facility RDW participants, their husbands were the decision-makers. However, due to the husbands’ unavailability at the onset of illness, the women had to wait before visiting the facility. Husbands and relatives also tended to advocate for alternative sources of care before a woman was allowed to be seen by their TBA or physician.

#### Local understandings of the PP period

Women and their families did not view many of the postpartum illness symptoms as severe or life-threatening. *Kamzori (weakness)* and *bukhar (fever)* were widely reported symptoms, but on their own, neither was considered worthy of care, particularly not from a physician.

#### Trust in services

Trust in available services depended on prior experiences and word-of-mouth. One respondent who had been seeing a doctor at a hospital prior to receiving care at JPMC blamed her illness on the treatment of her previous doctors: “*I have been taking care from the hospital throughout 8 months of pregnancy from a specialist doctor. However, she never said I had any health-related issues in any of the follow-ups, so I was satisfied with the treatment being given at the facility. It was her negligence; that’s why my condition got worse.*” Respondents mentioned that they would prefer to seek treatment at private facilities rather than government or welfare hospitals. People felt that they would receive better care at the former.

### Phase II: identifying & reaching care

#### Awareness and opting of different care sources

RDWs trusted and followed TBAs recommended by mothers-in-law and senior female relatives. Participants identified many sources of care, including local chemists (untrained shop owners dispensing antibiotics over the counter without prescription), faith healers, and local ‘clinics’ where paramedic staff (working in tertiary care facilities during the day) prescribed and dispensed medicine during evening hours. Participants were familiar with tertiary care facilities but said that they were only for worsening health conditions when the aforementioned sources did not provide adequate care.

Table 1 shows the different paths that RDW participants took while seeking care and whom they sought care. Each RDW clinically diagnosed with PP sepsis took a different path before reaching the tertiary care facility, JPMC. Two of the women sought care from faith healers where they received *taweez* or *purri* (small packets containing Quranic verses written on small pieces of paper” and *dam darood,* which is an herbal medicine for spotting during pregnancy and jaundice. Another respondent used burnt coconut to cure her vaginal discharge.

**Table 1:** Pathways of care-seeking among recently delivered women (RDW)

Pathway of care-seeking	Number of respondents (n = 10)
Home (doctor)	2 (20%)
Home (TBA)	1 (10%)
Home →clinic	1 (10%)
Home →clinic →TBA	1 (10%)
Home →clinic →hospital	1 (10%)
Home →clinic →welfare hospital →JPMC	2 (20%)
Home →private clinic →faith healer →JPMC	1 (10%)
Home →hospital → faith healer →JPMC	1 (10%)

Senior family members also gave *sadqa* (an act of charity or sacrifice) to relieve the RDW’s ailment. Respondents who used these alternative treatments reported some improvements in their symptoms after use.

#### Delayed referral to tertiary care facilities

Physicians knew that women sought care from many places and saw the tertiary care facility as a last resort:

*“Jinnah Hospital is a tertiary care hospital where they receive complicated referred cases. Most of them have been seeking care from TBAs, local practitioners, and untrained providers, and after worsening, they reach the tertiary setup. Therefore, they are probably very sick when they reach the tertiary care hospital. They have been delivered through untrained and unskilled providers with no aseptic measures taken, so usually women came with worse septic shock”. [Physician, JPMC 31–05].*

#### Financial access

Community and facility RDW were primarily poor; they had large families, prior loans, and small incomes. Women cited financial access as a barrier to facility care but not to the purchasing of medications. One woman went to Saylani Welfare, an organization that provides aid for people without the funds to go to a private hospital. A relative of an RDW at the facility discussed how finances had an impact on their decision to go to a government versus private hospital and how the family felt about this decision now:

*“Her husband had a two lacs loan and due to financial instability, we could not afford private facility expenses this time, so we chose a government hospital. She [the RDW] was not at all willing to get hospitalized in a government setup, but we were so helpless and there was no other option left. Now I think that we made a big mistake”*

In the community, none of the respondents reported finances as a barrier and said that if they received a referral from a TBA but could not afford the facility, they would be able to borrow sufficient funds.

#### Geographical/transport access

Women did not report problems with geographic access. While women did not live near facilities, they felt they could easily find a taxi, rickshaw, or ambulance to take them to the appropriate facilities.

### Phase III: receipt of adequate and appropriate care

#### Available resources

Women who managed to reach the health facility received acute medical care in the hospital at the time of labor and delivery. Women also sought medical attention for the management of complications, including prolonged and preterm labor pains, emergency cesarean section, rupture of the amniotic bag, retained placental pieces, sepsis, and increased vaginal bleeding post-delivery followed by hypotension and hypovolemic shock. All women who were diagnosed with PP sepsis were delivered to the hospital, where they received acute medical treatment. Later, they were transferred to the tertiary care facility.

#### Alternate sources of care in the community and self-treatment practices

For those RDWs in the community, many received advice and treatment from relatives, TBAs, chemists, and unlicensed providers. The perception of care quality was surprisingly good—most women who received care from spiritual healers believed that their symptoms had been relieved. Family members often believed that medication would be sufficient and adequate to relieve the woman of her symptoms. Typically, a male family member was sent to a provider close to home, described the woman’s symptoms, and purchased the medicine or treatment offered. Common treatments included allopathic medicines from medical stores and from medically trained providers as well as treatments from spiritual healers. Most women who were diagnosed with PP sepsis had already used some antibiotics before arriving at the facility. These had been purchased from chemists for the presumptive treatment of fever.

#### Case study

One female relative of a RDW diagnosed with PP sepsis discussed her daughter’s care-seeking behaviors:

Her daughter was admitted to JPMC (the government tertiary care facility) with an infection after her third delivery; the first two deliveries had been miscarriages or “spontaneous abortions”. To prevent a miscarriage in this latest pregnancy, the mother took her daughter to a faith healer who recited holy verses during all 9 months of pregnancy. The faith healer also gave the woman *taweez*, written holy verses to be worn as jewelry, to prevent spotting. The family was hoping for a male child, and the faith healers told them that due to their recitations of the Quranic verses, they would have a new baby boy in the family. The woman successfully carried the child to term but delivered a baby girl instead.

After the fourth postpartum day, a physician came around during a routine check-up and discharged the RDW. Soon after, the woman began experiencing a high-grade fever and was re-admitted to JPMC with a septic wound from her c-section. The relatives were not pleased that they needed to return to JPMC, especially since they would have preferred to go to a private clinic. Unfortunately, the husband had two loans and could not afford private facility expenses at this time. After the RDW had been readmitted, the family felt they had made a big mistake and should have chosen a private facility anyway. The RDW felt a lot of stress because of the finances and believed she was partially unwell due to the stress.

After receiving treatment for her infection, the physicians advised the woman to postpone her next pregnancy and start family planning. The husband agreed to this, but the woman felt too much pressure to deliver a baby boy to the family; she did not want her husband to seek a second marriage. This example illustrates the co-existence of cultural (trust in faith healing over modern medicine due to limited understanding, pressure to conceive and deliver a male child despite inadequate recovery), financial (dependence on male caretakers leading to waiting for symptoms to self-resolve), and quality care (surgical wound site infections leading to further distrust in facility-based care) barriers, all culminating in poor obstetric outcomes.

## Discussion

**Fig. 2:**
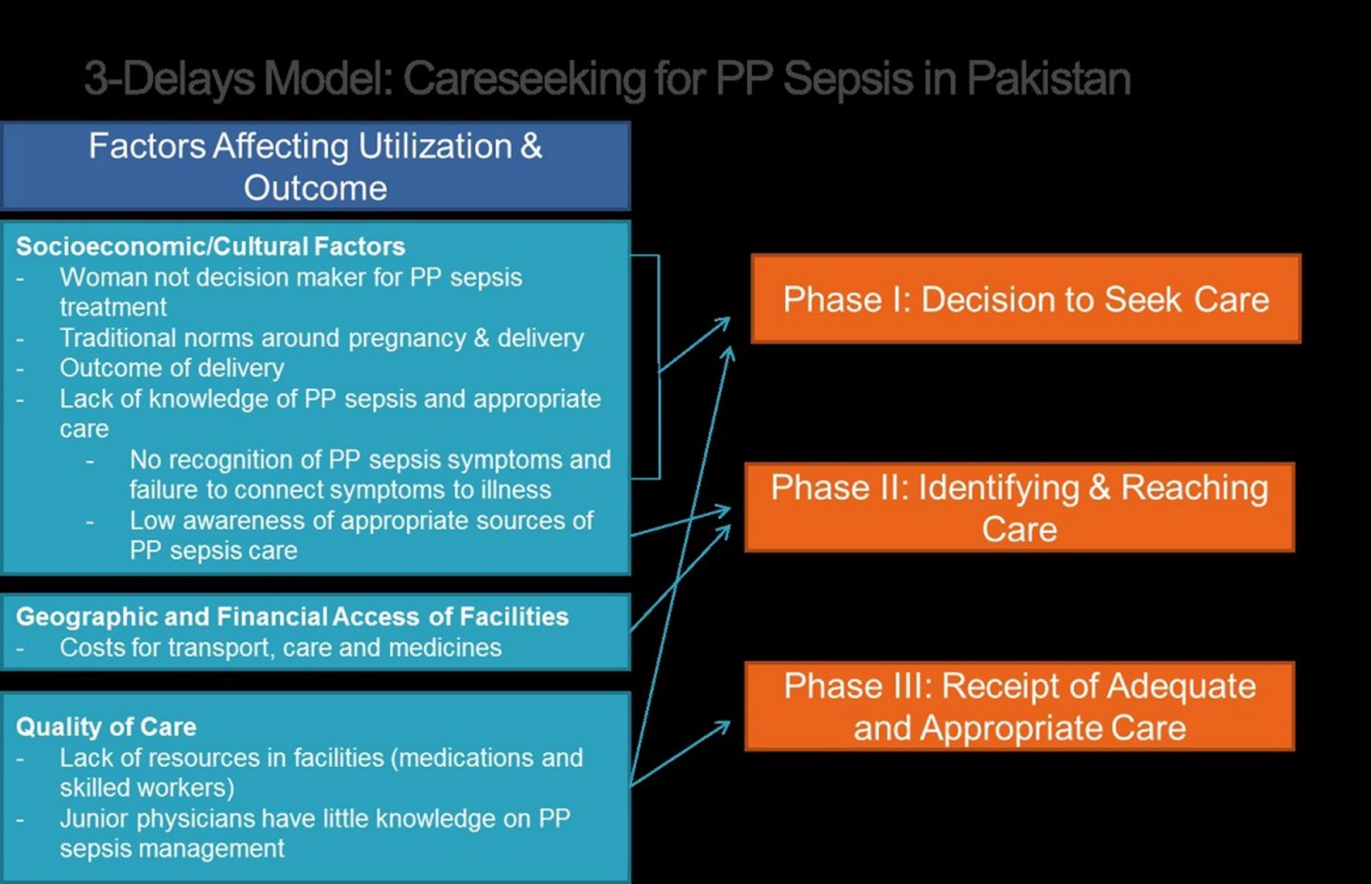
Three-delays model: care-seeking for postpartum sepsis in Karachi, Pakistan

This study explored the care-seeking patterns in RDWs with PP sepsis via a three-delay model. Figure 2 depicts factors responsible for the delay in women’s care-seeking for this life-threatening problem (Fig. 2).

Phase 1 indicated that women’s knowledge about PP sepsis was minimal, and they did not tend to view its symptoms as serious enough to seek care promptly. A systematic review based on 37 studies highlighted that inadequate knowledge of obstetric danger signs was one of the most important reasons for delay in care-seeking [20]. This may be because some women might perceive the danger signs and symptoms post-delivery to be normal and underestimate their severity, leading to delays in seeking medical attention. Moreover, studies have also shown that women with no formal education often have limited knowledge about post-delivery complications and thus don’t resort to seeking early tertiary care [21, 22].

One of the largest barriers to seeking care for PP sepsis, as found by our study findings, is women’s autonomy in their own decision-making. Several studies showed consistency with this finding that women usually lack autonomy to make decisions and were required to obtain permission from family members, particularly husband to access healthcare which causes delay in care seeking [14, 23, 24]. In Karachi, family members, particularly senior relatives, are often involved inevaluating the seriousness of the illness. If they do not consider it life-threatening, the woman is usually not permitted to seek care. In a community where terms for symptoms can have many meanings, the interpretation of seriousness can vary widely.

Findings of this study also indicated that women preferred alternate sources of care which included TBA’s, faith healers and chemist/health workers in local clinics rather than seeking care in a tertiary care facility. Studies show alignment with this finding suggesting that women and their families believed that post-delivery/obstetric complications are caused by witchcraft, which needs faith or traditional healers to resolve rather than allopathic medicine [21, 24]. Additionally, few women seemed to believe that obstetric complications resolve on their own and would prefer going to TBAs for alternate medications. Women also resort to chemists and untrained shop owners who distribute medicine without prescriptions which is extremely problematic. The World Health Organization recently released a report on global antimicrobial resistance that indicates that the overuse and misuse of antibacterial drugs has led to decreased effectiveness and, in some cases, complete ineffectiveness [25]. Efforts to increase the use of appropriate services will need to tackle the misuse of antibiotics.

Additionally, for the women and families in our study, poverty was an important barrier in finding and reaching an appropriate healthcare facility. While many participants did not specifically cite financial access as a problem, they did talk about not being able to leave their large families with no one else to care for the children. One RDW was only able to go to a welfare hospital because she could not afford private care. Families also discussed the effect of prior loans on their care-seeking choices. Literature is in agreement with this finding as it was found that women with low household income income were more likely to delay care seeking [20].

The Lady Health Worker (LHW) program in Pakistan has been shown to have a substantial positive impact on family planning, antenatal care, neonatal check-ups, and immunization, particularly in poorer households. There are several current LHWs and young women in Karachi who can be trained to screen and identify postpartum illnesses, including puerperal sepsis, and report to a physician (even with basic undergraduate training) at the catchment clinic or the closest secondary-level health facility. Most communities, especially in the Aga Khan University Department of Pediatrics research sites, have a clinic that can serve as a coordination and referral hub for women suspected of sepsis in identifying early and subtle signs of puerperal sepsis within the RDW's place of residence. Early screening and timely referrals will help to reduce the use of inappropriate sources of care and reduce morbidity and mortality associated with PP sepsis in Pakistan.

Other key barriers causing delays in care-seeking included lack of knowledge, minimal family support, financial constraints, and unavailability of transport. To address these, community-based education campaigns should be launched to spread awareness about PP sepsis signs and symptoms and its potential consequences, emphasizing early detection and timely care. These educational campaigns should include not only the pregnant women but also their families, especially male family members and head of household to ensure family support at the PP period. LHWs, along with stakeholders and community gatekeepers, should be involved in disseminating tailored and culturally appropriate information about PP sepsis for better receptivity. Maternal health packages should be devised that reduce out-of-pocket expenditure to as minimum as possible. Local health infrastructure, including clinics and BHUs, should be strengthened to ensure they are equipped for handling obstetric emergencies, reducing the need for long-distance transportation and its associated hefty costs. By tackling these barriers, prompt care can be sought for PP sepsis, ultimately improving maternal health outcomes.

## Conclusion

This study emphasizes that the delays in care-seeking for PP sepsis are primarily due to lack of awareness, financial constraints, cultural norms, and dependency on unskilled care and self-medication. To address these major issues, it is essential to have targeted community awareness sessions, improve accessibility to healthcare and integrate maternal healthcare services to community networks. Empowerment of women and their families and strengthening LHWs and CHWs can also aid in reducing the delays. Future interventions should work on reducing systemic delays to ensure that women receive life-saving healthcare without much delay to improve health outcomes.

## Supplementary Information

Supplementary material 1.

Supplementary material 2.

Supplementary material 3.

## Data Availability

The data that support the findings of this study are available from the Department of Pediatrics, Aga Khan University. Restrictions apply to the availability of these data, as they are not publicly available. Data are available upon request to the corresponding author.

## Abbreviations

PP: Postpartum
TBAs: Traditional birth attendants
CHWs: Community Health Workers
IDIs: In-depth interviews
RDW: Recently delivered women
LHW: Lady Health Worker
