# Supplementary material for: Patterns of care-seeking for postpartum symptoms in urban Karachi, Pakistan: implications for intervention design
Source: Reprod Health. 2025 Apr 16;22:55. doi: 10.1186/s12978-025-01981-8 (PMC12004814; doi:10.1186/s12978-025-01981-8)

Supplementary figure 1: Map showing routes of a tertiary medical center JPMC (for in-depth interviews), Bilal colony (for community interviews) and Aga Khan University Hospital. Map was created using software named ArcGIS 10.2.2. In the map, road distance and route was created from Bilal Colony to Hospitals using Network Analyst Extension in ArcGIS.


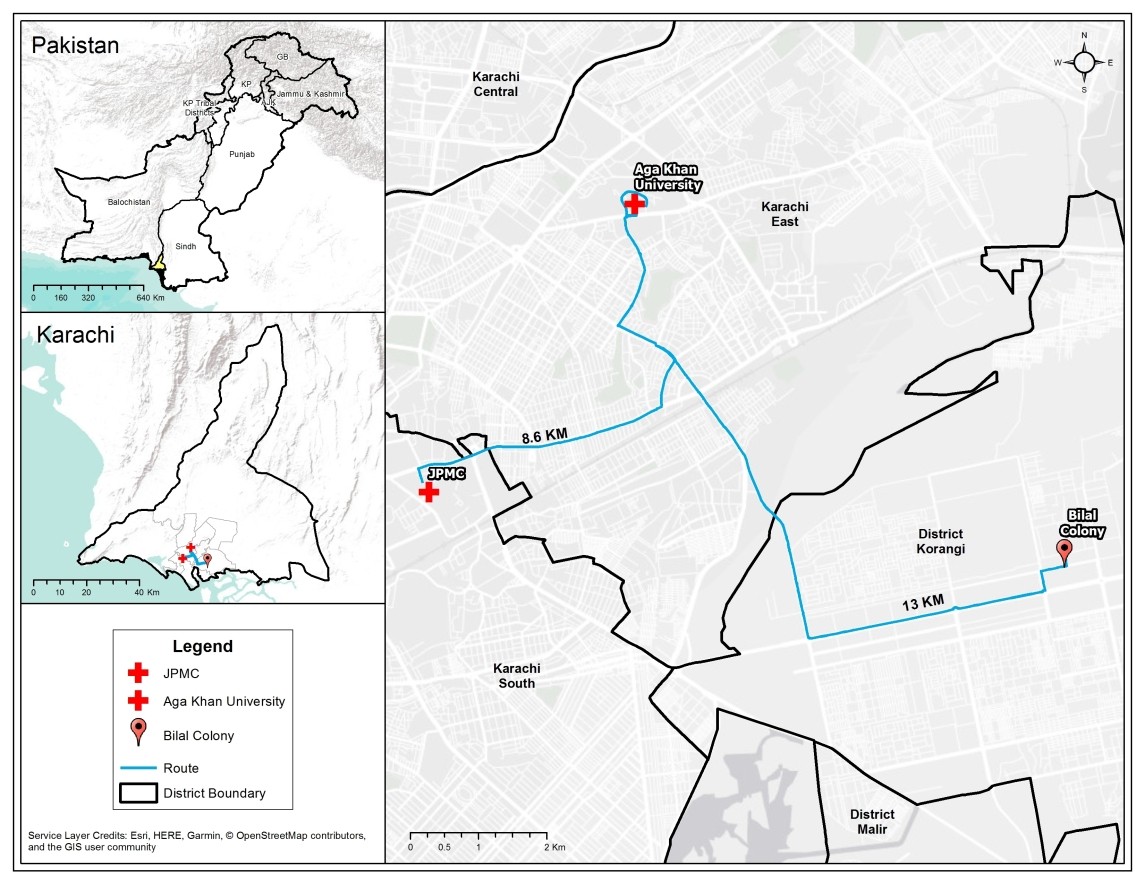

Supplement: Supplementary file 1 — Supplementary material 1. [file 12978_2025_1981_MOESM1_ESM.docx]
