# Supplementary material for: Patterns of care-seeking for postpartum symptoms in urban Karachi, Pakistan: implications for intervention design
Source: Reprod Health. 2025 Apr 16;22:55. doi: 10.1186/s12978-025-01981-8 (PMC12004814; doi:10.1186/s12978-025-01981-8)
Supplement: Supplementary file 2 — Supplementary material 2. [file 12978_2025_1981_MOESM2_ESM.pdf]

| 1 | | PP Sepsis |
|---|---|---|

Interviewer Name: ________________________
**Supervisor Id:** ________________________
**Transcriber Name:** ________________________ **Date of Transcription (DD/MM/YYYY):** ___________
**Translator Name:** ________________________ **Date of Translation:** ________________________

# Data Collection Instrument 1:
## Formative Study: Interview with Facility based Health Provider

**Form Version Date:** Version 2, 11 June2012
**JHU IRB:** 00004229
**___ IRB:**

**Instructions to Interviewer:** You will conduct a semi-structured interview with facility based health provider. Before starting the interview, be sure to take informed consent from the respondent. The goal of this interview is to: 1) determine their perceptions of the clinical signs and symptoms associated with women they have seen with postpartum sepsis; 2) gain an understanding of how they diagnose postpartum sepsis; and 3) gather information on how many woman visit the facility with postpartum illness and the signs and symptoms they report.

| 1. Address and identification information | | | | | | | | | | | | | |
|---|---|---|---|---|---|---|---|---|---|---|---|---|---|
| 1.01 | Respondent's Name | | | | | | | | | | | | |
| 1.02 | Respondent's designation | | MBBS?<br>Other? (specify___________________) | | | | | | | | | | |
| 1.03 | Name of the facility | | | | | | | | | | | | |
| | | | | | | | | | | | | | |
| 1.04 | District | | | | | | | | | | | | |
| 1.05 | Upazila | | | | | | | | | | | | |
| 1.06 | Union | | | | | | | | | | | | |
| 1.07 | Village | | | | | | | | | | | | |
| | | | | | | | | | | | | | |
| 1.08 | Date of Visit | | | d | d | | m | m | | y | y | y | Y |
| 1.09 | Check to confirm consent from respondent for conducting interview | | | | | | | | | | | | |

**Interviewer state:** As explained in the consent form, in our study we are trying to gain a better understanding of illnesses of women during the puerperal period (after birth until 42 days later). This interview will take about an hour to be completed.

01 For how long have you been providing services in this facility? ______ months

02 What are the common illness you found (within your catchment area) among postpartum (within 42 days after birth) women? For each illness, tell us at what point in the postpartum period it is most common, e.g. first week postpartum, second week postpartum.

______________________________________________________________________________

______________________________________________________________________________

______________________________________________________________________________

03 What are the common symptoms that women report to you who suffered postpartum (within 42 days after birth) sickness?

______________________________________________________________________________

______________________________________________________________________________

______________________________________________________________________________

04 What are the common symptoms you hear from postpartum women which trigger your thinking of puerperal sepsis?

______________________________________________________________________________

______________________________________________________________________________

______________________________________________________________________________

______________________________________________________________________________

05 Which symptoms do you think are very closely related to the diagnosis of puerperal sepsis?

______________________________________________________________________________

______________________________________________________________________________

______________________________________________________________________________

______________________________________________________________________________

06 When you examine a postpartum woman with fever who has sought care from you, what different diagnoses do you consider?

______________________________________________________________________________

______________________________________________________________________________

______________________________________________________________________________

___________________________________________________________________________

07 In case once you think of puerperal sepsis for a woman, how do you proceed to confirm the diagnosis of puerperal sepsis?

___________________________________________________________________________

___________________________________________________________________________

08 Is there any relevant data/record available about how many women sought treatment from this facility for any sickness/complaint during their postpartum period (from giving birth to a baby until 42 days after delivery) during last one year? Yes/ No

09 (To Interviewer) : If any such data/record available – Please copy the data from the record/register with permission from concerned authority

For which calendar year, the data is available? _________

What is the source of the data? _________________________ (write the name of the register)

| Month | Number of women attending the Outpatient clinic (OPD) with any complain during postpartum period | What are the recorded complaints for what women attended the OPD?<br><br>(Copy and List all the complaints you find recorded in the register) |
|---|---|---|
| January | | |
| February | | |
| March | | |
| April | | |
| May | | |
| June | | |
| July | | |
| August | | |
| September | | |
| October | | |

| | | |
|---|---|---|
| November | | |
| December | | |

10 Is there any relevant data/record available about how many women were admitted in this facility for any sickness/complaint during their postpartum period (from giving birth to a baby until 42 days after delivery) during last one year? Yes/ No

11 (To Interviewer) : If any such data/record available – Please copy the data from the record/register with permission from concerned authority

For which calendar year, the data is available? _________

What is the source of the data? _________________________ (write the name of the register)

| Number of women by month who got admitted in this facility with any complaint during postpartum period | What are the recorded complaints for what women were admitted?<br><br>(Copy and List all the complaints you find recorded in the register) | What are the recorded complaints/symptoms for a woman who was diagnosed as having puerperal sepsis at the time of admission?<br><br>(Copy and List all the complaints/symptoms from women who were diagnosed as having puerperal sepsis at the time of admission) |
|---|---|---|
| January=<br>February=<br>March=<br>April=<br>May=<br>June=<br>July=<br>August=<br>September=<br>October=<br>November= | | |

| | | |
|---|---|---|
| December= | | |

12 We are exploring how to identify women with postpartum sepsis more effectively at the community level, and get them to care more quickly. Do you have any general thoughts about how identify these women?

__________________________________________________________________________

__________________________________________________________________________

__________________________________________________________________________

__________________________________________________________________________

13 Do you have any thoughts about how to get women with postpartum sepsis the care they need more quickly?

__________________________________________________________________________

__________________________________________________________________________

__________________________________________________________________________

__________________________________________________________________________

14 We are trying to develop a clinical algorithm for use at community level. We intend to use the algorithm by community health workers to identify women suffering from puerperal sepsis. We would appreciate your any relevant comment, suggestion, feedback regarding listing signs and symptoms which could have potential benefit to develop such an algorithm.

__________________________________________________________________________

__________________________________________________________________________

__________________________________________________________________________

__________________________________________________________________________

Thank you so much taking the time to talk to me today.

Interviewer Name: ________________________
**Supervisor Id:** ____________________________
**Transcriber Name:** _________________________ **Date of Transcription (DD/MM/YYYY):** ___________
**Translator Name:** __________________________ **Date of Translation:**

## DATA COLLECTION INSTRUMENT 2:
## FORMATIVE STUDY: INTERVIEW WITH WOMAN AT FACILITY

**Form Version Date:** Version 2, 11 June 2012
**JHU IRB:** 00004229
**___ IRB:**

**Instructions to Interviewer:** You will conduct a semi-structured interview with postpartum women (within two months of giving birth) who have been admitted in this health facility with the diagnosis of puerperal sepsis (confirm with attending doctor/nurse). Before starting the interview, be sure to take informed consent from the respondent woman and copy answers from consent form for questions 1.08 to 1.11. The goal of this interview is to: 1) Gather illness and experiences narrative from the intrapartum and postpartum time frame and gain an understanding of terms for signs and symptoms used; 2) Interview about the evolution of the illness; 3) Identify careseeking decision making processes. Remember that the woman does not have to answer all questions and can quit at any stage.

| **1. Address and identification information** | | | | | | | | | | | | | |
|---|---|---|---|---|---|---|---|---|---|---|---|---|---|
| 1.01 | Woman's Name | | | | | | | | | | | | |
| 1.02 | Husband's Name | | | | | | | | | | | | |
| 1.03 | District | | | | | | | | | | | | |
| 1.04 | Upazila | | | | | | | | | | | | |
| 1.05 | Union | | | | | | | | | | | | |
| 1.06 | Village | | | | | | | | | | | | |
| 1.07 | Date of Visit | | | d | d | | m | m | | y | y | y | Y |
| 1.08 | Check to confirm consent from the respondent for conducting interview | | | | | | | | | | | | |
| 1.09 | Check to confirm consent from respondent to contact family member | | | | | | | | | | | | |
| 1.10 | Check to confirm consent from respondent to contact health provider | | | | | | | | | | | | |
| 1.11 | Check to confirm consent from respondent to view medical records | | | | | | | | | | | | |

**Interviewer State:** As explained in the consent form, in our study we are trying to gain a better understanding of your illness. I would like you to feel open to express yourself and feel free to expand

upon the experiences you are describing. This conversation will remain confidential. This interview will take about an hour to be completed.

15 Please recall and tell me, the first day of last menstrual period (before your last/recent pregnancy) [dd/mm/yy] ________________________________

16 How long was your pregnancy? _____________ month (as mentioned by the woman)

17 How did the pregnancy end? 1= Live birth

2 = Still birth

3 = Miscarriage/Abortion

If the woman delivered live birth or still birth, ask the following questions:

18 When (date) did you deliver your last child? (dd/mm/yy) ________________________

19 How was the baby delivered? 1= Normal/vaginal delivery,

2= Caesarian section,

3= Assisted delivery (Vacuum extraction/Episiotomy)

20 Where did you deliver? 1= At home

2= In this health facility,

3= In another health facility

4= Other (Please specify_________________________)

If the woman delivered at home or in any place other than health facility, please ask the following:

21 Who delivered the baby? 1= Family member

2= Traditional Birth Attendant,

3= Health Worker from nearby facility (FWV, Nurse, Paramedics)

4= Community Health Worker, NGO Health worker

5= Others (Please specify_________________________)

22 Was the baby a girl or a boy? 1 = Girl 2 = Boy

Interviewer: Now we are going to ask you a series of questions about your current illness, how you or your family recognized that you were sick and in need of treatment, and how you decided to come here

for treatment. We will begin with the illness you experienced after delivery and before coming here to this health facility.

23 When do you think the illness that brought you here to this health facility started? Did it start before you went into labor, during labor and delivery, or after delivery?

______________________________________________________________________________

24 Did you notice any problems or symptoms before you went into labor? What did you notice?

______________________________________________________________________________

______________________________________________________________________________

______________________________________________________________________________

25 Did you notice any problems or symptoms during labor and delivery? What did you notice?

______________________________________________________________________________

______________________________________________________________________________

______________________________________________________________________________

26 Now, can you please describe the illnesses and experiences you had after this pregnancy ended?

______________________________________________________________________________

______________________________________________________________________________

______________________________________________________________________________

______________________________________________________________________________

______________________________________________________________________________

**(The following questions can be used to help probing for information on the illness experience.)**

10 What signs or symptoms did you experience (make a sequential list)?

11 **Probe:** How long did each of these symptoms last? (Record the time period for each.)

12 Can you please describe how each of these felt and what you remember thinking or feeling about having this symptoms?

**13** **Probe:** What level of severity would you say there was for each of the symptoms? Please describe the level of pain or perceived severity. **Probe:** Were you able to conduct daily activities? Were their

sharp pains? If you feel comfortable, please explain in detail. Please indicate if you would categorize each symptom as not at all serious, serious, and very serious and why? **(Interviewer please categorize in notes.)**

14 **Probe:** Did the symptoms improve?

15 What is the name of illness you had? Can you please describe what the meaning of that is (it can be a local meaning)?

16 How were you able to identify the illness?

17 What do you believe caused this illness?

18 Can you please tell me more about the experiences surrounding this illness?

19 Did you use any type remedy or medicine (whether purchased, borrowed, or homemade)? [other than medicines taken at this facility]

20 If so, what did you use, when, and for how long? (ask what they used before and after seeking help, if they did seek help)

21 Did you seek help for these symptoms (before coming to this facility)?

22 From whom did you seek help?

23 Where (what are the places/persons) did you seek care from (excluding this facility)?

24 If you did not seek any help or care before coming to this facility, what reason did you have for not seeking any help or care?

25 If you did seek help, what were the circumstances those lead you to seek help to address these symptoms? What were your reasons for seeking help?

26 Did anyone encourage seeking help? If so, who? Was any other woman present? If so what was her name? Did any of your family members or neighbors encourage you to seek care for your sickness after delivery? If yes – who is she? Her name? Your relationship with her? Is she present here today? Would you feel comfortable if we asked her about your experiences as well?

**Interviewer state:** We also are interested to know about the care/treatment you received from all the facilities or health care delivery points you visited after you had these sickness/health problems (and before coming to this facility.)

27 What other different places/persons did you seek care from (for these sicknesses/health problems, except this facility)?

______________________________________________________________________________

______________________________________________________________________________

______________________________________________________________________________

28 For each of the people/places/facilities you visited (except this facility) – please tell us in detail the sequence of events and your experiences:

________________________________________________________________________________

________________________________________________________________________________

________________________________________________________________________________

________________________________________________________________________________

________________________________________________________________________________

________________________________________________________________________________

________________________________________________________________________________

________________________________________________________________________________

29 What were the recommendations/suggestions and treatment you received from all the places and persons you visited for your sicknesses? Please tell us sequentially all the treatments recommended to you.

30 Did you follow the suggestions/recommendations you received from those persons/places you visited?

31 (If not) Why?

________________________________________________________________________________

________________________________________________________________________________

________________________________________________________________________________

________________________________________________________________________________

32 Did you recover?

33 (If not recovered) Did the illness or sickness return?

34 If it did, please describe what you did after it returned.

________________________________________________________________________________

________________________________________________________________________________

________________________________________________________________________________

**Interviewer state:** Now before concluding, please tell us in detail, how did you end up at this facility? Tell us the sequence of events that led you to come to this facility. Who referred you here? What was the sequence of care and treatment you received here?

___________________________________________________________________________

___________________________________________________________________________

___________________________________________________________________________

___________________________________________________________________________

___________________________________________________________________________

___________________________________________________________________________

___________________________________________________________________________

___________________________________________________________________________

___________________________________________________________________________

___________________________________________________________________________

___________________________________________________________________________

___________________________________________________________________________

___________________________________________________________________________

___________________________________________________________________________

___________________________________________________________________________

___________________________________________________________________________

___________________________________________________________________________

___________________________________________________________________________

Thank you so much taking the time to talk to me today.

**Interviewer:** Check whether respondent has given consent to contact a family member. If she has given consent to this item, explain that we would like to talk to someone who was in close contact with her during her illness, preferably a senior female in the household.

35 Record names of 1 or 2 people who we can contact and details on how best to get in touch with these people.

______________________________________________________________________________

______________________________________________________________________________

______________________________________________________________________________

______________________________________________________________________________

______________________________________________________________________________

**Interviewer:** Check whether respondent has given consent to contact her health provider. If she has given consent to this item, explain that we would like to talk to the health provider (or TBA) who delivered her or provided care before she came to the facility.

36 Record names of 1 or 2 people who we can contact and details on how best to get in touch with these people.

______________________________________________________________________________

______________________________________________________________________________

______________________________________________________________________________

______________________________________________________________________________

______________________________________________________________________________

**Interviewer:** Check whether respondent has given consent to review her medical records. If she has given consent to this item, use this space to record important details of her case (i.e. time of admission, condition at admission and during later evaluations, symptoms and duration, lab results, treatment, etc.)

37 Notes from medical records.

______________________________________________________________________________

PP Sepsis ☐☐ - ☐☐

معلومات جمع کرنے والا سوال نامہ 1 : صحت کی سہولت فراہم کرنے والے کے ساتھ انٹرویو۔

PP Sepsis | **1**

انٹرویو لینے والے کا نام : ____________________

سپروائزر کا شناختی نمبر : ____________________ سپروائزر کا نام ____________________

تصدیق کنندہ کا نام(Transcriber): ____________________ تصدیق کی تاریخ ____/____/______ (DD/MM/YYYY)

# DATA COLLECTION INSTRUMENT 1:
## FORMATIVE INTERVIEW WITH FACILITY BASED HEALTH PROVIDER

**Form Version Date:** Version 4,25 July 2012

**JHU IRB:** 00004229

**AKU IRB : 2223 - Ped - ERC - 12**

انٹرویو لینے والے کیلئے ہدایت : - آپ ایک کلینک/ہسپتال میں طبّی خدمات مہیا کرنے والے سے انٹرویو لیں گے یہ فرد کو طبی تربیت یافتہ شخص ہو سکتا ہے جو کہ زچگی کے بعد ہونے والے انفیکشن کا علاج کرنے کا تجربہ رکھتا ہو۔انٹرویو شروع کرنے سے پہلے اس بات کی یقین دہانی کرلیں کہ جواب دہندہ سے اجازت لے لی گئی ہے۔ اس انٹرویو کا مقصد : - بچے کی پیدائش کے بعد خواتین میں زچگی کے بعد ہونے والے Sepsis کی طبّی علامات/نشانیوں کے شعور کا پتہ لگانا ہے : - (2) زچگی کے Sepsis کے بارے میں بتاتے ہوئے خواتین جو الفاظ استعمال کرتی ہیں اس کا پتہ لگانا: - (3) یہ سمجھنا کہ بچے کی پیدائش کے بعد کے Sepsis کی کیسے تشخیص کرتے ہیں۔

فارم کی تیاری :ٹرمنالوجی فارم "A" سوال نمبر 03,02 اور 04 ہیلتھ ورکر کے لئے علیحدہ سے کاپیاں بنائیں اور فری لسٹنگ کرتے ہوئے بھریں۔آپ ٹرمنالوجی فارم B بھی بھریں گے اپنے نوٹس کی مدد سے انٹرویو ختم ہونے کے بعد

فری لسٹ مشق کیلئے ہدایات: فری لسٹ کا سوال دوبارہ سے مختلف لفظوں میں مت پوچھیں۔انٹرویو دینے والے کی یاداشت کو ہلکے پھلکے دھکے لگائیں،مثلاً ٹھیک ہے،آپ نے بخار کا ذکر کیا، اور کوئی شکایت ہوئی؟ ٹھیک ہے آپ نے سر درد اور کمزوری کا ذکر کیا اسکے علاوہ اور کو مسئلہ درپیش ہوا۔

پتہ اور شناختی معلومات :

| | | | | |
|---|---|---|---|---|
| | | | جواب دہندہ کا نام | 1.01 |
| | | ایم بی بی ایس<br>دیگر وضاحت کریں ____________ | جواب دہندہ کا عہدہ | 1.02 |
| | | | کلینک کا نام | 1.03 |

طبی سہولت فراہم کی جانے والی جگہ :

| | | |
|---|---|---|
| | ملک سائٹ | 1.11 |
| | ضلع | 1.12 |
| | تالوکہ/(ٹاؤن) | 1.13 |
| | یونین کونسل | 1.14 |
| | گاؤں (ٹاؤن شپ) | 1.15 |

معلومات جمع کرنے والا سوال نامہ 1 : صحت کی سہولت فراہم کرنے والے کے ساتھ انٹرویو۔

| وزٹ کی معلومات : | | |
|---|---|---|
| 1.21 | وزٹ کی تاریخ | d d m m y y y y |
| 1.22 | کیا آپ نے تصدیق کر لی کہ اس انٹرویو کیلئے جواب دہندہ نے رضا مندی دی ہے۔ | |

**انٹرویو لینے والا باآواز پڑھے** : - جیسا کہ اجازت نامہ میں بتایا گیا ہیکہ ہم اپنے مطالعہ میں بچے کی پیدائش کے بعد (پیدائش سے 42 دن بعد تک) ماؤں میں Sepsis (بچے دانی کی انفیکشن) کو بہتر طور پر سمجھنے کی کوشش کر رہے ہیں۔ ہمارے مطالعہ کا مقصد آپکے علاقے میں ماؤں میں بچے کی پیدائش کے بعد Sepsis کی شناخت اور جلد ہی ان کا علاج کرنے میں مدد کرنا ہے صحت مہیا کرنے والے افراد سے بات کرنے کا مقصد یہ ہے کہ ہم بہتر طریقے سے سمجھ سکیں کہ مائیں بچے کی پیدائش کے بعد ہونے والے Sepsis کو کیسے اور کن الفاظ میں بیان کرتی ہیں۔ اس کے علاوہ ہم اُس عمل کو بھی سمجھنے کی کوشش کر رہے ہیں جو کہ اُس عورت کے ساتھ جو کہ بچے کی پیدائش کے بعد Sepsis کا شکار ہے ہسپتال میں جا کر ختم ہوتا ہے۔ اس انٹرویو کو ختم ہونے میں تقریباً ایک گھنٹہ لگے گا۔

01 آپ اس کلینک/ہسپتال میں کب سے خدمات انجام دے رہی ہیں۔ ____________________ (مہینے ، سال)

**سوال نمبر 2 اور 3 کا مقصد کسی بھی قسم کی بیماری/علامات کا بیان جو کہ مائیں زچگی کے بعد بتاتی ہیں اس کو درج کرنا ہے تا کہ ہم زچگی کے بعد ہونے والے انفیکشن کو بہتر سمجھ سکیں**

02 فری لسٹ : - زچگی کے بعد مائیں اور ان کے خاندان بچے کی پیدائش کے پہلے 42 دنوں کے دوران کون سے عام صحت کے مسائل آپ سے بیان کرتے ہیں؟ ہم اُن مخصوص الفاظ کو سننا چاہیں گے جو کہ مائیں اور اُن کے خاندان صحت کے مسائل کو بیان کرنے کیلئے استعمال کرتی ہیں نہ کہ میڈیکل ٹرمنر۔

**Q.2 کا ٹرمنالوجی فارم A پر جواب لکھیں**

02.1 اب بتائیں کہ ہر بیماری/علامات جو آپ نے اوپر درج کروائی ہے مائیں اور ان کے خاندان والے کب ان شکایات کا بتاتے ہیں۔ مثال کے طور پر بچے کی پیدائش کے بعد پہلے ہفتے میں یا دوسرے ہفتے میں۔

**Q.2.1 کا جواب ٹرمنالوجی A فارم میں سیدھے ہاتھ کے کالم میں لکھیں**

03 **فری لسٹ** : - وہ مائیں جو بچے کی پیدائش کے بعد کسی بیماری میں مبتلا ہوتی ہیں وہ ان علامات کو بتانے کیلئے عام طور پر کیا الفاظ استعمال کرتی ہیں؟ ہم پھر ایک دفعہ اُن الفاظ کو سننا چاہیں گے جو کہ مائیں اور ان کے خاندان زچگی کے بعد کے صحت کے مسائل کو بیان کرنے کیلئے استعمال کرتی ہیں نہ کہ میڈیکل ٹرمز۔

**Q.3 کے جوابات ٹرمنالوجی فارم A کی 2nd کاپی پر لکھیں**

**Q.4 بچہ کی پیدائش کے بعد کے ماں کے Sepsis کے بارے میں پوچھنا ہے؟**

04 **فری لسٹ** : - آپ کے خیال میں وہ کون سی عام علامات اور مسائل ہیں جو کہ جب بچے کی پیدائش کے بعد آنے والی خواتین بتاتی ہیں تو آپ سمجھتے ہیں کہ اسکا نتیجہ زچگی کے Sepsis کی صورت میں نکل سکتا ہے یہ جوابات ٹرمنالوجی فارم A کی 3rd کاپی پر لکھیں۔

معلومات جمع کرنے والا سوال نامہ 1 : صحت کی سہولت فراہم کرنے والے کے ساتھ انٹرویو۔

**Q.4 کے جوابات ٹرمنالوجی فارم A کی 3rd کاپی پر لکھیں**

4.1 اوپر درج کی گئی ہر علامات کیلئے، برائے مہربانی یہ بھی بتائیں کہ بیماری کی علامات کتنی شدید ہونگی تو آپ حمل کے بعد ہونے والے عورتوں کے Sepsis کا سوچیں گی۔ کتنی شدت کی نوعیت ہوگی تو آپ کہیں گی کہ یہ اب قدرتی صحت یابی کا حصّہ نہیں بلکہ قابل علاج ہے۔

**Q.4.1 کا جواب Q4 کے ٹرمنالوجی فارم A کے چوتھے کالم میں لکھیں۔**

**Q.5 بچے کی پیدائش کے بعد ماں کو ہونے والے بخار یا ممکنہ زچگی کے بعد کے Sepsis کے بارے میں پوچھے گا**

05 جب آپ بچے کی پیدائش کے بعد آنے والی عورت جس کو بخار ہے کو دیکھ رہے ہوتے ہیں تو آپ کیا مختلف تشخیص (Differential Diagnoses) پر توجہ دیتے ہیں۔

__________

06 جب آپ یہ سوچتے ہیں کہ ماں کو زچگی کے بعد کا Sepsis ہے تو اس تشخیص کی تصدیق کیلئے آپ کیا لائحہ عمل اختیار کرتے ہیں۔ (How do you Confirm Daignosis)

__________

**سوال 7 سے 9 کیلئے صحت کی سہولت فراہم کرنے (Health Care Provider) والے فرد کی حوصلہ افزائی کریں کہ وہ اپنے خیالات اور تجاویز بتائیں۔**

07 ہم اس بات کا پتہ لگانے کی کوشش کر رہے ہیں کہ ہم کس طرح اور بہتر طریقے سے آپ کے علاقے میں اُن ماؤں کو تلاش کرسکیں جن کو بچے کی پیدائش کے بعد کا Sepsis ہے تا کہ وہ جلد از جلد علاج کرواسکیں۔ آپ کے خیال سے اس علاقے میں ان ماؤں کی شناخت (How to identify) اور کیسے کی جاسکتی ہے (مثال کے طور پر ان کے گھروں میں)۔

__________

08 آپ کے خیال میں کمیونٹی میں ہم اُن ماؤں تک کیسے پہنچ سکتے ہیں 1 ان کو کیسے ڈھونڈ سکتے ہیں جن کو بچے کی پیدائش کے بعد Sepsis ہے (مثال کے طور پر اُن کے گھروں میں) مقصد اُن کو وہ طبّی امداد دی جلد از جلد دیتا ہے جس کی ان کو ضرورت ہے۔

09 ہم ایسا سوالنامہ بنانے کی کوشش کر رہے ہیں جو کہ کمیونٹی ہیلتھ ورکر بچے کی پیدائش کے بعد ہونے والے گھروں کے وزٹ کے دوران اُن عورتوں کو ڈھونڈنے میں استعمال کرے گی جن کو بچے کی پیدائش کے بعد Sepsis ہوسکتا ہے ہم آپ کی ان علامات اور نشانیوں کے بارے میں رائے، مشوروں اور فیڈ بیک کی قدر کرتے ہیں جو کہ اس قسم کے سوالنامے کی تشکیل میں فائدہ مند ہونگے۔

مجھے وقت دینے کا بہت بہت شکریہ

**For interviewer: mark if form completed:**

Question 2, Terminology Form A ______

Question 3, Terminology Form A ______

Question 3, Terminology Form A ______

Terminology Form B completed for this questionnaire? ______

PP Sepsis | 2

| | |
|---|---|
| انٹرویو لینے والے کا نام : ____________ | |
| سپروائزر کا شناختی نمبر : ____________ | سپروائزر کا نام ____________ |
| تصدیق کنندہ کا نام (Transcriber) : ____________ | تصدیق کی تاریخ ____/____/____ (DD/MM/YYYY) |

# DATA COLLECTION INSTRUMENT 2:

## FORMATIVE INTERVIEW WITH POSTPARTUM SEPSIS WOMAN ADMITTED IN A FACILITY

**Form Version Date:** Version 4,25 July 2012

**JHU IRB:** 00004229

**AKU IRB : 2223 - Ped - ERC - 12**

آپ یہ انٹرویو اُن ماؤں سے لیں گیں جن کے ہاں بچے کی پیدائش دو مہینے کے اندر ہوئی ہو اور جو وضع زچگی کے Sepsis کی تشخیص کے ساتھ ہسپتال میں داخل ہیں PP Sepsis کی تشخیص دیکھنے والا ڈاکٹر زبانی کر سکتا ہے۔ انٹرویو لینے سے پہلے اس بات کا یقین کرلیں کہ جواب دہندہ ماں سے اجازت لی جا چکی ہے اس فارم میں سوال نمبر 1.08 سے 1.11 کے احتیاط سے لکھیں اس انٹرویو کا مقصد ہے 1 : - زچگی اور زچگی کے بعد کے عرصے میں بیماریوں اور تجربات کو اکھٹا کرنا تا کہ علامات اور نشانیاں کو بیان کرنے کیلئے جو الفاظ استعمال کئے جاتے ہیں اُن کو سمجھا جا سکے۔ 2 : - بیماریوں کے انکشاف یا بڑھاؤ کے بارے میں انٹرویو۔ 3 : - صحت کی سہولت تک پہنچنے کے فیصلے کے عمل کو سمجھنا۔ یاد رکھیں کہ ماں کے پاس سارے سوالات کے جوابات نہیں ہونگیں اور وہ کسی بھی مرحلہ پر انٹرویو ختم کر سکتی ہے۔

فارم کی تیاری : - ٹرمنالوجی فارم B کی کاپی بنائیں انٹرویو ختم کرنے کے بعد اس انٹرویو سے نوٹس لے کر اس کو مکمل کریں۔

پتہ اور شناختی معلومات :

| | | |
|---|---|---|
| | عورت کا نام | 1.01 |
| | شوہر کا نام | 1.02 |
| | ہسپتال/کلینک کا نام | 1.03 |

طبی سہولت فراہم کی جانے والی جگہ :

| | | |
|---|---|---|
| | ملک/سائٹ | 1.11 |
| | ضلع | 1.12 |
| | اُپزلا/تعالقہ | 1.13 |
| | یونین کونسل | 1.14 |
| | گاؤں/ٹاؤن شپ | 1.15 |

وزٹ کی معلومات :

| | | | | | | | | | | |
|---|---|---|---|---|---|---|---|---|---|---|
| d | d | m | m | y | y | y | y | | وزٹ کی تاریخ | 1.21 |
| | | | | | | | | | کیا آپ نے چیک کر لیا ہے کہ اس انٹرویو کیلئے ماں سے اجازت لے لی گئی ہے۔ | 1.22 |

PP Sepsis [ ][ ] - [ ][ ]

معلومات جمع کرنے والا سوال نامہ 2 : PP Sepsis میں مبتلا عورت سے انٹرویو۔

**انٹرویو لینے والے کا بیان** جیسا کہ اجازت نامہ میں بتایا گیا ہے کہ ہم اپنے اس مطالعہ میں آپ کی بیماری کو بہتر طریقے سے سمجھنا چاہتے ہیں۔ میں چاہوں گی کے آپ پوری طرح کُھل کر اپنے تجربات کو بیان کریں ہماری یہ بات چیت صیغہ راز رکھی جائیں گی اس انٹرویو کو مکمل ہونے میں تقریباً ایک گھنٹہ لگے گا۔

01 برائے مہربانی یاد کریں اور مجھے بتائیں کہ آپ کی آخری ماہواری کا پہلا دن کونسا تھا (آخری/ حالیہ حمل سے پہلے) ____/____/______ (dd/mm/yyyy)

02 آپ کا حمل کتنی مدت کا تھا؟ ______ مہینے (جیسا کہ ماں بتائے)

03 اس حمل کا نتیجہ کیا تھا؟

1۔ زندہ بچہ

2۔ مردہ بچہ

3۔ بچہ ضائع ہو گیا (اگر جواب دہندہ بچہ ضائع ہونے کا جواب دے تو یہاں انٹرویو ختم کر دیں اور اس کا شکریہ ادا کریں)

**سوال نمبر 4 سے 8 میں ماں سے اس کی زچگی کے بارے میں پوچھیں، جو کہ آخری دہ ماہ میں ہوئی ہے۔**

04 آپ کی آخری زچگی/ بچے کی پیدائش کب ہوئی؟ ____/____/______ (dd/mm/yyyy)

05 بچے کی پیدائش کس طرح ہوئی؟

1۔ نارمل وجائنل ڈلیوری

2۔ آپریشن (Caesarian section),

3۔ چھوٹا آپریشن (Vacuum extraction/Episiotomy)

06 بچہ کی پیدائش کہاں ہوئی؟

1۔ گھر پر (سوال نمبر 7 پر جائیں)

2۔ اسی جگہ ہسپتال/ کلینک میں (سوال نمبر 8 پر جائیں)

3۔ کسی اور کلینک/ ہسپتال پر (سوال نمبر 8 پر جائیں)

4۔ کوئی اور (وضاحت) ______________________

**اگر ماں کی زچگی یا بچہ کی پیدائش گھر پر ہوئی ہے یا کلینک/ ہسپتال کے علاوہ کہیں اور ہوئی ہو تو برائے مہربانی مندرجہ ذیل سوالات پوچھیں۔**

07 بچہ کی پیدائش کس نے کروائی؟

1۔ گھر کے فرد

2۔ دائی

3۔ قریبی طبی سہولت (نرس مڈوائف، لیڈی ہیلتھ ورکر، لیڈی ہیلتھ وزیٹر)

4۔ کمیونٹی ہیلتھ ورکر، NGO ہیلتھ ورکر

5۔ کوئی اور وضاحت کریں ______________________

PP Sepsis ☐☐ - ☐☐

معلومات جمع کرنے والا سوال نامہ 2 : PP Sepsis میں مبتلا عورت سے انٹرویو۔

08 بچہ لڑکا تھا یا لڑکی؟ 1۔ لڑکی
2۔ لڑکا

**سوال نمبر 9 سے 11 تک حمل سے پہلے، اس کے دوران اور بعد کے بارے میں پوچھیں۔**

انٹرویو لینے والی : - اب میں آپ سے آپ کی حالیہ بیماری کے بارے میں سوالات کروں گی آپ اور آپ کی فیملی نے کب یہ جانا کہ آپ بیمار ہیں اور آپ کو علاج کی ضرورت ہے، اور آپ نے یہاں آ کر علاج کروانے کا فیصلہ کیا۔ ہم آپ کی اُس بیماری سے شروع کریں گے جو کہ یہاں آنے سے پہلے اور زچگی کے بعد آپ کو ہوئی۔

09 زچگی کے درد میں جانے سے ہفتے یا مہینے پہلے کیا آپ نے اپنی حالیہ بیماری سے متعلق مسائل کے بارے میں کبھی نوٹس کیا، اگر ہاں، تو کیا نوٹس کیا؟

____________________

10 کیا زچگی کے دردوں کے دوران آپ نے اپنی حالیہ بیماری سے متعلق مسائل کا نوٹس لیا؟ اگر ہاں تو آپ نے کیا نوٹس لیا؟

____________________

11 بچے کی پیدائش کے بعد کے دن یا ہفتوں میں آپ نے اپنی حالیہ بیماری سے متعلق مسائل کے بارے میں نوٹس لیا؟ اگر ہاں تو کیا؟

____________________

معلومات جمع کرنے والا سوال نامہ 2 : PP Sepsis میں مبتلا عورت سے انٹرویو۔

سوال نمبر 12 اس ہسپتال میں داخل ہونے سے پہلے کے تمام واقعات سے ہے جو ایک کے بعد دوسرے ترتیب سے ہوئے۔

12 آپ حالیہ بیماری کے شروع سے لے کر اس وقت تک جب تک آپ ہسپتال میں داخل ہو گئی کا واقعہ تفصیل سے بیان کریں؟

**متبادل سوال : -** آپ نے صحت کا کونسا پہلا مسئلہ نوٹس کیا؟ اور اس کے بعد کونسا مسئلہ نوٹس کیا؟ اس کے بعد کیا ہوا؟ یہ تب تک پوچھیں جب تک جوابدہندہ یہ نہ بتا دیں کے کس مسئلہ کے بعد اس نے کلینک/ہسپتال کا رخ کیا؟

معلومات جمع کرنے والا سوال نامہ 2 : PP Sepsis میں مبتلا عورت سے انٹرویو۔

**سوال 13 : - (13.1 سے 13.4) زچگی کے بعد ہونے والی عورتوں کے Sepsis کے بارے میں سوالات ہیں۔**

13 **ترتیب وار لسٹ :** - آپ نے کیا تکلیف/ علامات محسوس کیں۔ آپ پر کیا تکلیف بیماری کی علامات گزریں ہیں؟

**انٹرویور : - نیچے دیئے گئے ٹیبل میں لکھیں ( کالم 13 علامات کا نام )**

13.1 کیا آپ ہر علامت کو تفصیلی طور پر بیان کر سکتی ہیں؟

**انٹرویور : - نیچے دیئے گئے ٹیبل میں لکھیں ( کالم 13.1 جو بتایا گیا ہے )**

13.2 ہر علامت کیلئے کیا آپ بتا سکتی ہیں کہ ہر علامت کتنے عرصے رہی (ہر ایک کا وقت گھنٹوں، دنوں میں لکھیں) ؟

**انٹرویور : - نیچے دیئے گئے ٹیبل میں لکھیں ( کالم 13.2 دورانیہ )**

13.3 ہر علامت کیلئے کیا آپ بتا سکتی ہیں کہ وہ کتنی شدید تھی یا کس نوعیت کی تھی، برائے مہربانی یہ بھی بتائیں کہ آپ نے کتنی تکلیف یا شدّت کا درجہ محسوس کیا؟

**متبادل سوال** : - ہر علامت کیلئے، کیا آپ نے محسوس کیا تھا کہ یہ بہت شدید تھا، یا آپ نے محسوس کیا کہ یہ معمولی شدت کا ہے، جب آپ بہت شدید یا اور معمولی شدت کہتی ہیں تو اس سے آپکا کیا مراد ہے،

**متبادل سوال** : - کیا آپ روزانہ کے معمولات انجام دینے کے قابل تھیں؟

**انٹرویور : - نیچے دیئے گئے ٹیبل میں لکھیں ( کالم 13.3 شدت )**

13.4 ہر صحت کے مسئلہ کے لئے بیان کریں، کیا اس میں بہتری ہوئی، ہاں یا نہیں ؟

**انٹرویور : - نیچے دیئے گئے ٹیبل میں لکھیں ( کالم 13.4 بہتری )**

| سوال 13 علامات کے نام | سوال 13.1۔ تفصیل (اگر جگہ کافی نہ ہو تو لائین کے نیچے لکھیں) | سوال 13.2۔ دورانیہ (دنوں میں) | سوال 13.3۔ شدت | سوال 13.4۔ بہتری ہاں / نہیں |
|---|---|---|---|---|
| | | | | |
| | | | | |
| | | | | |
| | | | | |
| | | | | |
| | | | | |

PP Sepsis ☐☐ - ☐☐

معلومات جمع کرنے والا سوال نامہ 2 : PP Sepsis میں مبتلا عورت سے انٹرویو۔

| Q.13۔ علامات کے نام | Q 13.1۔ تفصیل (اگر جگہ کافی نہ ہو تو لائین کے نیچے لکھیں) | Q.13.2۔ دورانیہ (دنوں میں) | Q13.3۔ شدت | Q 13.4۔ بہتری ہاں / نہیں |
|---|---|---|---|---|
| | | | | |
| | | | | |
| | | | | |
| | | | | |
| | | | | |
| | | | | |
| | | | | |

**سوال نمبر 14 سے 17 بچے کی پیدائش کے بعد کا Sepsis کی کیفیت/ بیماریوں کی شناخت کے بارے میں ہے۔**

14 آپ کو جو بیماری تھی اُسکا نام کیا ہے، کیا آپ بتا سکتی ہیں کہ اس کا مطلب کیا ہے (علاقائی زبان میں مطلب اور بھی ہو سکتا ہے)؟

**اگر جوابدہندہ جواب نہیں دے سکتا، یہ وہ معلومات لکھیں جو جوابدہندہ دے سکے اور سوال نمبر 15 پر چلے جائیں۔**

______________________________

______________________________

______________________________

______________________________

______________________________

15 آپ نے اس بیماری کو کیسے شناخت کیا؟

______________________________

______________________________

______________________________

______________________________

______________________________

16 کس نے یہ پہچانا کہ آپ بیمار ہیں؟ یعنی، کیا آپ نے پہچانا کہ یہ مسئلہ ہے، یا رشتہ داروں نے، پڑوسی نے، دائی نے یا کوئی اور تھا؟

17 آپ کے خیال میں اس بیماری کی وجہ کیا تھی؟

**سوال نمبر 18 علاج کے بارے میں ہے**

18 آپ نے کیا علاج اور نسخہ استعمال کئے تھے؟

**پوچھیں** : - کیا آپ نے مساج، گرم کپڑے، اور دوائیں (چاہے خریدی ہو، ادھار لی ہوں یا گھر کی بنی ہوئی ہوں) کا استعمال کیا یہاں ہسپتال میں آنے سے پہلے؟ آپ نے یہ کب تک استعمال کیں، کس نے خریدیں،

**انٹرویور، نیچے دئے گئے ٹیبل میں لکھیں۔**

ہاں ________ نہیں ________

اگر مدد لینے سے پہلے کوئی اگر کوئی نسخہ یا علاج نہ استعمال کیا ہو تو ______ **سوال نمبر 19 پر جائیں۔**

| نسخہ/علاج کا نام | استعمال کا دورانیہ | کیسے خریدی تھیں؟ |
|---|---|---|
| | | |
| | | |
| | | |
| | | |
| | | |
| | | |
| | | |
| | | |

PP Sepsis ☐☐ - ☐☐

معلومات جمع کرنے والا سوال نامہ 2 : PP Sepsis میں مبتلا عورت سے انٹرویو۔

**سوال نمبر 19 سے 27 تک صحت کی سہولت کی رسائی حاصل کرنے کے سلسلے میں درپیش مشکلات کے بارے میں ہیں۔**

19 اس ہسپتال/کلینک میں آنے سے پہلے آپ نے اس بیماری کیلئے کسی سے مدد لی؟

ہاں ________

نہیں ________ اگر نہیں تو 23 پر چلے جائیں

20 اگر آپ نے مدد لی تو اس مدد کو لینے کی وجہ کیا تھی؟

________________________________________

________________________________________

________________________________________

21 اگر آپ نے مدد لی تو، اس بات کا اخری فیصلہ کس نے کیا کہ کب اور کہاں سے مدد لی جائے؟ فیصلہ کرنے والے فرد کا نام اور اس سے آپ کا رشتہ کیا ہے؟ کیا وہ اس وقت یہاں ہے؟ آپ کو کوئی اعتراض نہ ہو تو ہم اس فرد سے بھی آپ کے تجربہ کے بارے میں کوئی سوال کریں؟

________________________________________

________________________________________

________________________________________

________________________________________

________________________________________

**انٹرویور کا بیان : - اس ہسپتال میں آنے سے پہلے آپ نے دیگر صحت کی سہولت مہیا کرنے والی جگہوں پر جتنے علاج/ دوائیں/ٹوٹکے استعمال کئے وہ خاص دلچسپی کا باعث ہیں۔**

22 اگر آپ دیگر جگہوں سے علاج کروایا یا دکھا کر آئے ہیں تو وہ کون سی مختلف جگہیں تھیں (نام بتائیں)؟

**پوچھیں** پہلی جگہ/فرد کیا تھی جہاں سے آپ نے صحت کی سہولت لی۔۔۔اس کے بعد کہاں گئی۔۔۔اس کے علاوہ۔۔۔پوچھنا جاری رکھیں جب تک وہ حالیہ صحت کی سہولت کا نہ بتادے۔

**انٹرویور : - نیچے دئے گئے ٹیبل میں اندراج کریں (کالم 22 فرد/صحت کی سہولت جہاں مریض نے وزٹ کیا)**

22.1 ہر اس فرد / جگہ / طبی سہولت جہاں آپ گئیں ان کے ساتھ آپ کا تجربہ کیسا رہا؟

**انٹرویور : - نیچے دئے گئے ٹیبل میں اندراج کریں (کالم 22.1 تجربات)**

PP Sepsis ☐☐ - ☐☐

معلومات جمع کرنے والا سوال نامہ 2 : PP Sepsis میں مبتلا عورت سے انٹرویو۔

22.2 اس بیماری کے لئے آپ جہاں جہاں گئیں براۓ مہربانی آپ ترتیب وار علاج بتائیں جو آپ کو تجویز کیا گیا۔ اس کے ساتھ دواؤں کی تفصیل بھی اس میں نیز طبّی علاج جیسا کہ مساج/گرم کپڑا لگانا شامل ہیں؟

| انٹرویور : - نیچے دۓ گئے ٹیبل میں اندراج کریں (کالم 22.2 علاج ) |
|---|

22.3 کیا آپ نے اُن تجویزوں/مشوروں پر عمل کیا جو کہ آپ کو ان جگہوں اور لوگوں سے ملیں؟ اگر نہیں تو کیوں؟

| انٹرویور : - نیچے دۓ گئے ٹیبل میں اندراج کریں (کالم 22.3 عمل کیا ) |
|---|

| سوال22۔ جگہ یا فرد جہاں مدد کیلئے گئے | سوال22.1 تجربات | سوال22.2 علاج | سوال22.3 عمل کیا اگر نہیں تو کیوں نہیں؟ |
|---|---|---|---|
| | | | |
| | | | |
| | | | |
| | | | |
| | | | |
| | | | |
| | | | |

23 اگر آپ نے اس ہسپتال/کلینک میں آنے سے پہلے کوئی مدد نہیں لی تو اُس کی وجہ کیا تھی؟

______________________________

______________________________

______________________________

______________________________

______________________________

معلومات جمع کرنے والا سوال نامہ 2 : PP Sepsis میں مبتلا عورت سے انٹرویو۔

24 انٹرویور کا بیان اب برائے مہربانی ہمیں تفصیل سے بتائیں، آپ اس سہولت تک کیسی پہنچی؟ آپ ان تمام حالات کو ترتیب وار بتائیں جو یہاں آنے کا سبب بنے؟ یہاں آپ کو کس نے بھیجا؟ اور وہ تمام علاج اور دیکھ بھال ترتیب وار بتائیں جو آپ نے یہاں حاصل کیں؟

25 آپ اس صحت کی سہولت کس سواری سے یعنی کیسے پہنچی؟ اور آپ نے یہاں تک پہنچنے کے لئے کیسی سواری استعمال کی؟

26 یہاں پر آنے کا فیصلہ کرنے اور بالآخر یہاں پہنچ جانے میں آپ کو کتنا عرصہ لگا؟ (منٹ، گھنٹے، دن)

معلومات جمع کرنے والا سوال نامہ 2 : PP Sepsis میں مبتلا عورت سے انٹرویو۔

27 اس کلینک/ہسپتال تک پہنچنے کیلیئے آپ کو مشکلات کا سامنا کرنا پڑا؟

نہیں ________

اگر ہاں :

کیا ٹرانسپورٹ ملنا مشکل تھی؟ ________________________

کیا علاج اور ٹرانسپورٹ کیلیئے پیسے ملنا مشکل تھے؟ __________________

کیا آپ کے ساتھ کوئی جانے والا ملنا مشکل تھا؟ __________________

کیا یہاں آنے کیلیئے اجازت ملنا مشکل تھا؟ ____________________

کوئی اور وضاحت کریں؟ ________________________

**سوال 28 سے 30 تک سوال افاقہ کے بارے میں ہیں**

28 کیا آپ کو افاقہ ہوا؟ ہاں اگر نہیں تو انٹرویو ختم کردیں؟

________________________________________

________________________________________

________________________________________

29 اگر افاقہ ہوا تو کیا بیماری ٹھیک ہونے کے بعد پھر ہوئی ؟

________________________________________

________________________________________

________________________________________

30 اگر دوبارہ ہوئی تو برائے مہربانی بتائیں کہ آپ نے کیا کیا؟

________________________________________

________________________________________

________________________________________

میں آپ کے صبر کی داد دیتی ہوں اور آپ کے جوابوں کیلیئے شکر گزار ہوں یہ معلومات ہمیں آپ کے علاقے میں وضع حمل زچگی کے Sepsis اصطلاحات یا ٹرمنالوجی کو معلوم کرنے میں مددگار ثابت ہونگیں اور یہ نہ صرف پورے ملک میں بلکے پوری دنیا میں زچگی کے بعد ہونے والے Sepsis کو جلدی تشخیص کرنے میں مددگار ثابت ہونگیں، آپ کے وقت اور کوشش کا بہت بہت شکریہ۔

| |
|---|
| انٹرویور کیلیئے : - نشان لگائیں اگر فارم مکمل ہے : |
| اس سوالنامے کا ٹرمنالوجی فارم B مکمل ہے ________________ |
