# Supplementary material for: Patterns of care-seeking for postpartum symptoms in urban Karachi, Pakistan: implications for intervention design
Source: Reprod Health. 2025 Apr 16;22:55. doi: 10.1186/s12978-025-01981-8 (PMC12004814; doi:10.1186/s12978-025-01981-8)
Supplement: Supplementary file 3 — Supplementary material 3. [file 12978_2025_1981_MOESM3_ESM.docx]

***Supplementary Table 1: Socio-demographic information of study participants***

|  | **Karachi (Facility)**  **N=14** | **Karachi (Community) N=18** | **Total** |
| --- | --- | --- | --- |
| **Age (Years)** | | | |
| - 18-35 | 9 | 10 | 19 |
| - 36-50 | 3 | 4 | 7 |
| - >50 | 2 | 4 | 6 |
| **Education** | | | |
| - Illiterate/can’t sign | 4 | 11 | 15 |
| - Class 1-5 | 3 | 1 | 4 |
| - Class 6-10 | 1 | 6 | 7 |
| - Class 11-15 | 0 | 0 | 0 |
| - Class > 16 | 6 | 0 | 6 |
| **Occupation** | | | |
| - Doctor | 6 | 0 | 6 |
| - TBA | 0 | 6 | 6 |
| - Housewife | 8 | 12 | 20 |
| - Others | 0 | 0 | 0 |
| **Settings and socio-economic status** | | | |
| - Orangi number 13 (slum area) | 1 | 0 | 1 |
| - Gizri village (slum) | 1 | 0 | 1 |
| - Karongi 5 coast guard (middle) | 2 | 0 | 2 |
| - Noorani Basti (slum area) | 1 | 0 | 1 |
| - Reti Lines Cantt (slum area) | 1 | 0 | 1 |
| - Bhens Colony (slum area) | 2 | 0 | 2 |
| - Ibrahim Haideri (slum area) | 0 | 1 | 1 |
| - Bilal colony (slum area) | 0 | 17 | 17 |
| **Religion** | | | |
| - Islam | 14 | 18 | 32 |
